# The “best practices for farming” successfully contributed to decrease the antibiotic resistance gene abundances within dairy farms

**DOI:** 10.3389/fvets.2024.1420282

**Published:** 2025-01-07

**Authors:** Barbara Salerno, Matteo Cornaggia, Raffaella Sabatino, Andrea Di Cesare, Claudio Mantovani, Lisa Barco, Benedetta Cordioli, Luca Bano, Carmen Losasso

**Affiliations:** ^1^Laboratory of Microbial Ecology and Genomics, Istituto Zooprofilattico Sperimentale delle Venezie, Legnaro, Italy; ^2^Microbiology and Veterinary Diagnostic Laboratory, Istituto Zooprofilattico Sperimentale delle Venezie, Villorba, Italy; ^3^Molecular Ecology Group (MEG), Water Research Institute-National Research Council of Italy (CNR-IRSA), Verbania, Italy; ^4^Science Communication Unit, Istituto Zooprofilattico Sperimentale delle Venezie, Legnaro, Italy

**Keywords:** antibiotic resistance, antimicrobial consumption, dairy farms, calves, antimicrobial resistance genes (AR genes)

## Abstract

**Introduction:**

Farms are significant hotspots for the dissemination of antibiotic-resistant bacteria and genes (ARGs) into the environment and directly to humans. The prevalence of ARGs on farms underscores the need for effective strategies to reduce their spread. This study aimed to evaluate the impact of a guideline on “best practices for farming” aimed at reducing the dissemination of antibiotic resistance.

**Methods:**

A guideline focused on prudent antibiotic use, selective therapy, and hygienic and immune-prophylactic practices was developed and provided to the owners of 10 selected dairy farms and their veterinarians. Fecal samples were collected from lactating cows, dry cows, and calves both before and after the implementation of the guideline. ARGs (*bla*_TEM_, *ermB*, *sul2*, and *tetA*) were initially screened by end-point PCR, followed by quantification using digital droplet PCR. ARG abundance was expressed in relative terms by dividing the copy number of ARGs by the copy number of the 16S rRNA gene.

**Results:**

The ARG abundances were higher in lactating cows compared to other categories. Despite similar levels of antibiotic administration (based on veterinary prescription data from the sampled farms) in both sampling campaigns, the total abundance of selected ARGs, particularly *bla*_TEM_ and *tetA*, significantly decreased after the adoption of the farming guidelines.

**Discussion:**

This study highlights the positive impact of prudent antibiotic use and the implementation of farming best practices in reducing the abundance of ARGs. The lactating cow category emerged as a crucial point of intervention for reducing the spread of antibiotic resistance. These findings contribute to ongoing efforts to address antibiotic resistance in farm environments and strengthen the evidence supporting the adoption of good farming practices.

## Introduction

Antimicrobial resistance is a threat to 21st-century environments, livestock production, and public health (1). Antibiotic resistance is driven by antibiotic resistance genes (ARGs), which are prevalent in environments where antimicrobial use exerts positive selective pressure (2), such as in farm environments. The selective pressure facilitates the acquisition of ARGs by bacteria through horizontal gene transfer (HGT), enabling them to spread within farm microbial communities and disseminate into the environment (3). Additionally, both bacteria harboring ARGs and ARGs in farming environments can enter the food chain, posing a serious risk to food safety and directly threatening farmers’ health. Livestock production, as a major consumer of antimicrobials, is recognized as a significant contributor to antibiotic resistance (4), leading to consumer concern about the use of antimicrobials in farming. In response, major meat distribution chains have focused on promoting and marketing “antibiotic-free” products or ensuring that no antibiotic treatments are administered during the final breeding period (5). This approach is not applicable in dairy farming due to its continuous production cycle and the high incidence of certain bacterial infections, such as those caused by *S. aureus* (6). For this reason, treating infections in dairy animals requires careful therapeutic decisions. Thus, to reduce drug resistance in dairy herds the current challenge extends beyond merely reducing antimicrobial use; it involves promoting alternative strategies such as improved farm management and more timely, accurate diagnostics. These measures aim to address both sustainable farming practices and public health concerns. While antibiotics should be used when necessary to treat sick animals, they must also be used prudently to preserve their effectiveness (7). Achieving these goals requires strengthening farmers’ knowledge of the antimicrobial resistance phenomenon and driving their perceptions regarding animal health, and consequently antibiotic use choices (8, 9).

Starting from these considerations, this study aimed to explore the potential for reducing the load of selected ARGs in dairy herds by increasing awareness among farmers and veterinarians, focusing on improvement of diagnostic strategies for recurrent bacterial infections and enhancing knowledge about the prudent use of antibiotics.

## Materials and methods

### Study design

We tested the efficacy of an awareness-raising and information campaign directed at farmers and farm veterinarians, aimed at reducing the prevalence of ARGs within the farm. The campaign focused on promoting: (i) Prudent use of antimicrobials, (ii) Adoption of selective therapy for dry cows, and (iii) Implementation of hygienic and immune-prophylactic practices. In each farm, a specific health problem was identified, and tailored measures were implemented to address it. These interventions were designed based on initial surveys and diagnostic results, ensuring they were adapted to the unique circumstances of each farm. Farm problems and corrective measures that were applied for a period of 2 years were reported in Table 1. The indicators of the effectiveness of these processes were (1) the trend of antimicrobial consumption throughout the project, calculated on the basis of an electronic veterinary prescription (10), which returns the data in “defined daily dose” (DDD), and (2) the spread of selected ARGs, quantitatively evaluated as ratio toward the entire resident microbial community of biological samples collected in the farms.

**Table 1:** Tailored guidelines drafted for the detection, diagnosis, control, and prophylaxis of meningitis, septicemia, neonatal enteric diseases, respiratory diseases, and mastitis.

Farm	n° bovines	Laboratory remarks	Critical issues evidenced	Action undertaken
A	185	Mastitis due to *S. aureus* and *Str. Agalactiae*	Impossibility to constitute separated milking groups (robot for milking in the farm)	Production and administration of a bivalent (*S. aureus, Str. agalactiae*) tailor-made vaccine
B	179	Neonatal enteritis due to *Rotavirus* and *Coronavirus*	Wrong vaccination timing for the prevention of calf diarrhea (*Rotavirus, Coronavirus, E. coli* K99) in pregnant bovines Suspension of the calf diarrhea vaccination in summer Irrational administration of antimicrobials for calf enteritis sustained by viruses	Correction of the vaccination protocols for the prevention of calf diarrhea Check of the correctness of the colostrum administration Suspension of the irrational antimicrobial treatments
C	139	Neonatal enteritis due to *Cryptosporidium* spp.	The water employed for the washing of the feed-buckets was contaminated by *Cryptosporidium* Feed-buckets not disinfected with products active against *Cryptosporidium* Irrational administration of antimicrobials for calf enteritis sustained by *Cryptosporidium*	Disinfection with products active against *Cryptosporidium* (creosol 4%) of the feed equipment for calves (25) Accurate dry of the feed-buckets after the cleaning procedures
D	248	Mastitis caused by *S. aureus* infection	Identification of lactating cows carrying *S. aureus*	Creation of milking groups according to the positivity to *S. aureus* in the milk Vaccination of the herd with a tailor-made vaccine
E	290	Blanked dry cow therapy	Absent circulation of contagious agents of mastitis Systematic administration of antimicrobials at the dry-off	Bacteriological examination as a routine procedure of the milk with high somatic cell count (SCC) Selective antimicrobial dry cow therapy based on the bacteriological and antimicrobial susceptibility test (AST) results
F	206	Coccidiosis in young heifers	Increase of the coccidia load in the feces of heifers placed in collective boxes Low frequency of the litter replacement Abuse of sulphonamides for coccidia treatments	Removal of the litter, cleaning with high pressure hot water and disinfection with product active against coccidia oocysts Increase of the frequency of the above mentioned actions
G	141	Neonatal enteritis due to *Rotavirus*	Low immunoglobulins titer in calves sera and colostrum (passive transfer failure)	Adoption of the right timing and procedures for the colostrum administration to the new-born subjects Creation of pooled colostrum stocks previously checked for immunoglobulin titer
H	248	Bovine respiratory disease in calves sustained by *Pasteurella multocida* and *Histophilus somni*	Lack of the aetiological diagnosis Irrational administration of antimicrobials to symptomatic subjects	Collection of bronco-alveolar lavages Virological and bacteriological examinations Therapy based on the bacteriological and AST results Adoption of a vaccine strategy based on the results
I	271	Mortality in calves due to Salmonella Dublin infection	The manure removal system conveyed the litter close to the calves hutches Carrier bovines were nor detected neither isolated Biosecurity failures Low number of farmworkers	Detection, grouping, isolation and progressive elimination of chronic carriers of S. Dublin Vaccination of the herd with a tailor-made vaccine Rational disposal of the calves hutches with respect to the manure removal
J	67	Circulation of bovine viral diarrhea virus (presence of persistent infected bovines)	Unspecific diseases in different animal categories Sporadic reproductive problems	Eradication program (26)

Ten dairy farms located in four different provinces (A, B, C, D, E, F, G, H, I, J) distributed in Northeastern Italy were visited over a 2 year period between January 2020 and June 2022. During the first visit, a farm questionnaire structured into 2 main parts was administered to farmers with the help of farm veterinarians. The first part was aimed at acquiring the general data regarding the farms, the productive and reproductive data, the number of housed animals divided by production phase, the breeds reared, the breeding premises, and the stabling system. The second part of the form was aimed at establishing the knowledge of the animal keeper regarding health issues strictly connected with the use of antibiotics such as (i) mastitis, (ii) neonatal enteritis, (iii) respiratory diseases, (iv) podal affections, and (v) circulation of BVD virus on the farm as a predisposing agent of bacterial diseases.

Farmers and veterinarians were aware of the risks of selecting antibiotic resistant microorganisms following the incorrect use of antibiotics and about the possibility of selecting the appropriate antibiotic drug and administering it at the appropriate dose, driven by the results of bacteriological investigations and of antimicrobial susceptibility tests (AST) based on the determination of the Minimum Inhibitory Concentration (MIC). In addition, with specific reference to the drying time, farmers and veterinarians were also taught about the opportunity of implementing the selective dry strategy consisting of rational therapies, avoiding systematic treatments of cows with antibiotics at drying-off. Moreover, starting from the experience gained in the field through empirical observations about the main infectious diseases of dairy cattle, a set of guidelines were drafted for the detection, diagnosis, control, and prophylaxis of meningitis, septicemia, neonatal enteric diseases, respiratory diseases, and mastitis (Table 1). Based on the guidelines, corrective actions were implemented, with particular reference to calf enteritis, such as the strict observance of the vaccination rules and the administration of immune colostrum to calves with prophylactic purposes. Finally, in order to mitigate the microbial contaminations among calves, farmers and veterinarians were encouraged to enhance the level of biosecurity in the calving period. This involved the manure removal frequency, thorough cleansing, and disinfection of calf hutches by using aerosol products (4% with a minimum contact time of 2 h) which have also shown efficacy against *Cryptosporidium* sp. (11). Finally, fecal samples were collected before (January 2020) and after (June 2022) the proposed corrective measures were adopted by farmers and veterinarians to determine whether these interventions effectively reduced the abundance of selected ARGs. Regarding farm J, as reported in Table 1, once all persistently infected (PI) individuals in the various animal categories (calves, heifers, dry cows, lactating cows) were identified and removed, all new calves born in the following 12 months were tested at birth for the presence of the virus in the ear cartilage. No vaccination protocol was implemented, but new animals were systematically tested before their introduction to the farm, and an annual monitoring program for BVD virus was established in the herd.

### Sampling scheme

Fecal samples were individually collected from lactating cows, dry cows, heifers (females aged 2 to 24 months), and calves (animals under 2 months old) as previously described (4). The sample size was determined according to the herd’s bovine population. In herds with more than 150 bovines, fecal samples were obtained from the rectal ampulla of 20 lactating cows, 10 dry cows, 10 heifers, and 5 calves (Supplementary Table 1). For herds with fewer than 150 bovines, individual fecal samples were collected from 15 lactating cows, 5 dry cows, 5 heifers, and 5 calves (Supplementary Table 1). Farms C, G, and H were categorized as small (<150 animals total), while farms A, B, D, E, F, I, and J were classified as large farms (≥150 animals total) (Supplementary Table 1). Samples were immediately transported to the laboratory under refrigeration conditions. Two sampling campaigns were carried out, the first one took place in 2020 (4) before the corrective measures were applied, and the second in 2022.

### Antibiotic consumption

Data on antimicrobial consumption for 2019 (the year before adopting the measures detailed in the guideline) and 2021 (2 years after the adopting the measures) in the sampled farms were accessible through the information system of the Italian Integrated Program for the Classification of Intensive Animal Farming (ClassyFarm), provided by the General Directorate of Animal Health and Veterinary Medicines of the Ministry of Health (12).

### Sample processing, DNA extraction and ARG detection and quantification

Fecal samples were grouped together in sets of five. Each composite sample was stomached for 1 min at room temperature in order to achieve homogenization. Three replicates were processed for each sample. A 0.2 mL volume was used for DNA extraction following the same procedure previously published (4). To enable the comparison of ARG presence and abundance before and after the implementation of the “best farming practices,” we selected the same ARGs previously analyzed (4). In detail, we chose *bla*_TEM_ and *bla*_CTXM_, which encode resistance to *β*-lactams; *qnr*S, responsible for quinolone resistance; *sul*2 and *tet*A as representative resistance genes against two of the earliest discovered and widely used antibiotics, sulfonamides and tetracycline, respectively; *erm*B, which encodes MLS resistance; *van*A, associated with glycopeptide resistance; and *mcr*-1, particularly significant at the clinical level as it encodes resistance against colistin, a last-resort antibiotic. The eight selected ARGs were firstly analyzed by end-point PCR and, where positive, they were quantified by digital droplet PCR (ddPCR) as already reported in Salerno et al. (4). The data were presented as gene copies per μL, and the analysis was conducted using QX Manager 1.2 (Bio-Rad). The abundance of ARGs was normalized by calculating the ratio of their copy number to the copy number of the 16S rRNA gene.

### Statistical analysis

The statistical analyses were conducted in the R environment v4.2.1 (13) to assess the impact on ARG abundance of implementing the measures described in the guidelines in farm activities. Prior to do this, we evaluated the dynamics of ARGs during the second sampling to verify whether the trends observed in the previous campaign were consistent. The differences in the normalized abundances of *bla*_TEM_, *erm*B, and *sul*2 genes were investigated, first, by MANOVA, analyzing genes collectively, and, then, by ANOVA (Tukey post-hoc tested), for the single genes. In both cases, abundances were prior transformed in the root square of the arcsine of their value, since they represent proportion data. The animal category (4 levels) and the farm (10 levels) were used as explanatory variables in the models. Difference in the total normalized abundance of ARGs were also evaluated by ANOVA with a Tukey post-hoc test, applying the same parameters. Afterwards, we compared, via ANOVA, the single (*bla*_TEM_, *erm*B, and *sul*2) and total normalized abundances measured in 2020 and those quantified in 2022, both dividing or not the samples per category. Also, the differences between the total and single antibiotics prescribed during the two sampling campaigns were tested by ANOVA.

### Correlation between ARG abundance and antibiotic consumption

The correlation between the normalized abundance of *bla*_TEM_, *erm*B and *sul*2 genes and the consumption of the corresponding antibiotic (i.e., penicillin, MLS, and sulphonamide) was assessed through Pearson’s correlation. The same test was done also in case of total normalized abundance of ARGs and total consumption of antibiotics. Variables were considered as correlated for *r* > 0.75 and *p* < 0.05.

## Results

### Antibiotic resistance gene presence, abundance, and antibiotic consumption

The ARG presence and abundance of the samples collected during the first sampling campaign (2020) were previously shown (4). Among the eight ARGs analyzed by PCR, only *bla*_TEM_, *erm*B, and *sul*2 genes gave a positive signal, at least, in one sample in the second campaign (Supplementary Table 2), for this reason, only these genes were quantified by ddPCR. Their abundances were comprised between 1.8 × 10^−5^ and 9.5 × 10^−2^ gene copies/16S rRNA gene copy (Supplementary Table 3). Specifically, *bla*_TEM_ was always quantifiable and its concentration ranged from 1.8 × 10^−5^ to 3.5 × 10^−3^ gene copies/16S rRNA gene copy; *sul*2 abundance was comprised between 2.2 × 10^−5^ and 9.5 × 10^−2^ gene copies/16S rRNA gene copy, being not detectable only in one sample (DC sample from D farm); *erm*B concentration ranged from 2.0 × 10^−5^ to 2.8 × 10^−2^ gene copies/16S rRNA gene copy and the gene resulted not detectable in two samples (H samples from F and H farms) (Supplementary Table 3).

**Figure 1:**
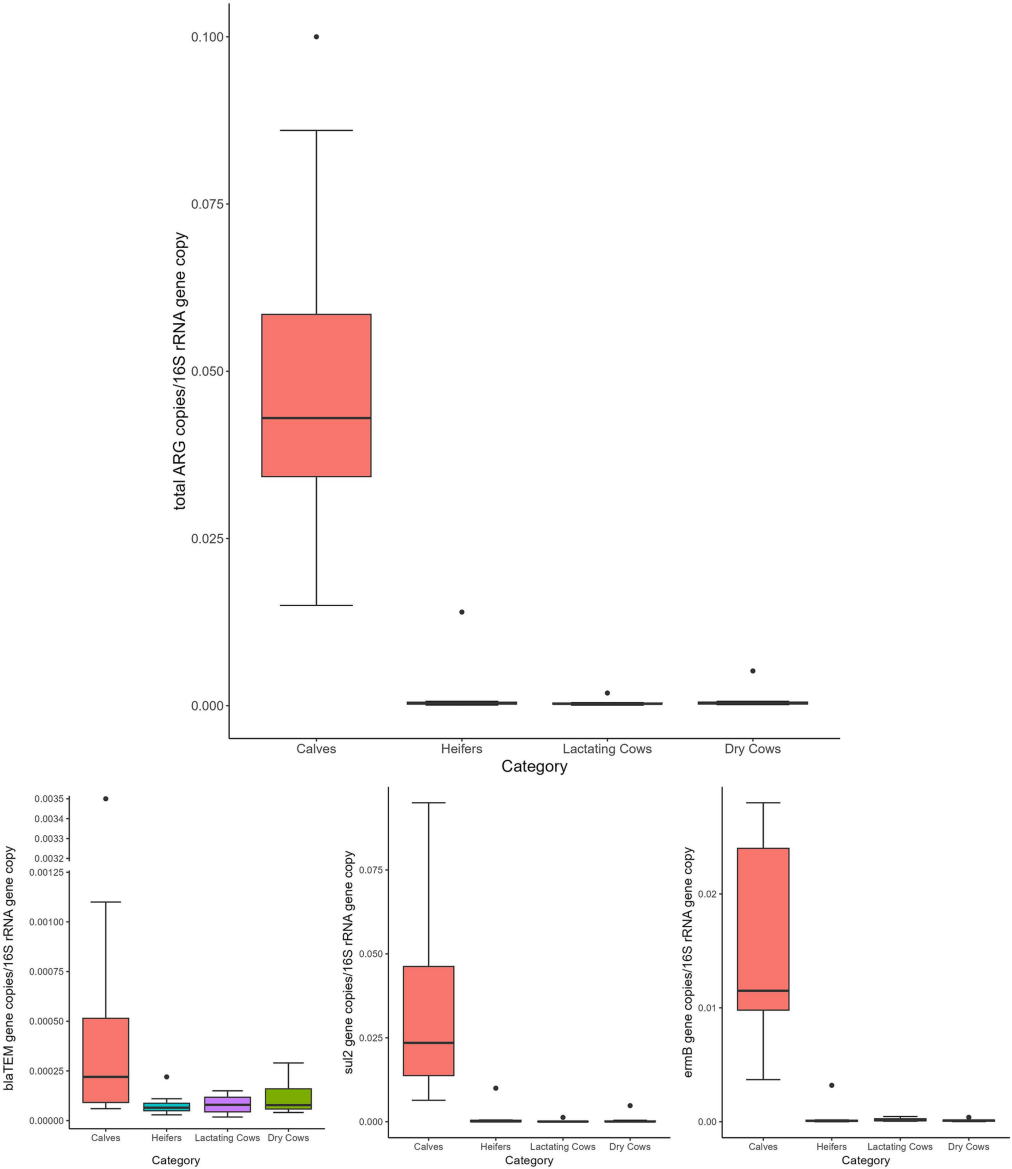
Normalized abundances of genes according to animal category. Boxplots of the distribution of abundances of total ARGs, *bla*_TEM_, *erm*B, and *sul*2 genes within the bacterial communities of calves, heifers, lactating cows and dry cows. The thick horizontal line represents the median, the box represents 50% of the values, the whiskers extend to the highest and lowest value within the 1.5 interquartile range, dots represent the outliers.

**Figure 2:**
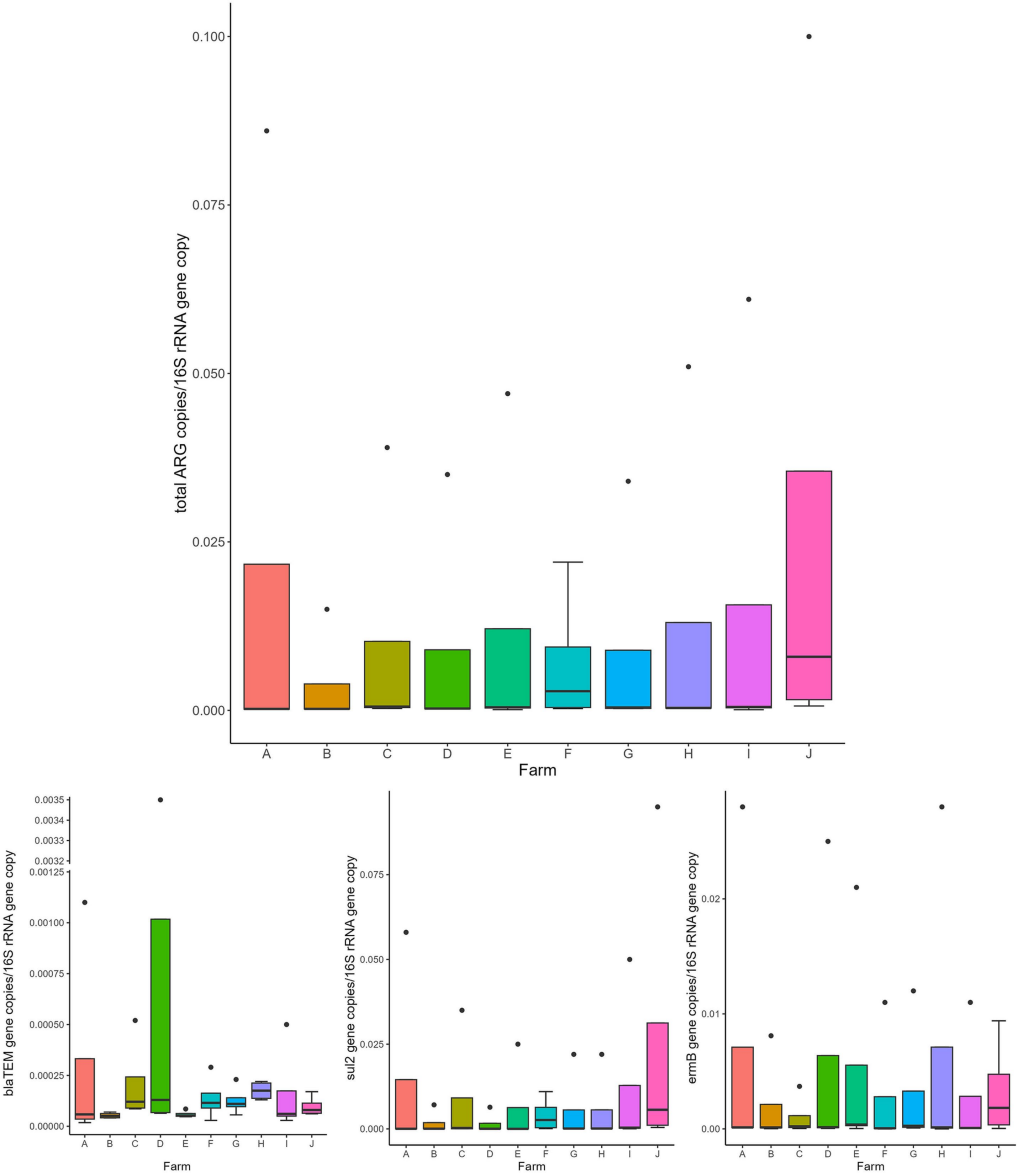
Normalized abundances of genes according to farm. Boxplots of the distribution of abundances of total ARGs, *bla*_TEM_, *erm*B, and *sul*2 genes within the bacterial communities of the investigated farms (A–J). The thick horizontal line represents the median, the box represents 50% of the values, the whiskers extend to the highest and lowest value within the 1.5 interquartile range, dots represent the outliers.

Overall, the normalized abundance of the tested genes significantly varied in relation to the animal category (MANOVA: *p* = 0.0002). Indeed, for both single and total ARGs, the abundance was significantly higher in calves than in the other farming steps (ANOVA: *p* ≤ 0.0105) (Figure 1 and Table 1). On the contrary, when considering the farm as statistical factor, no differences were observed (MANOVA: *p* = 0.2251), with similar ARG abundances among the samples (ANOVA: *p* ≥ 0.0698) (Figure 2 and Table 2).

**Table 2:** Statistical results for the analysis of variance (ANOVA) assessing the influence of the experimental variables (animal category and farm) on the normalized abundance of total ARGs, *bla*_TEM_, *erm*B, and *sul*2 genes.

	Df	Sum Sq	Mean Sq	*F*-value	*p*-value	*Post-hoc* grouping
**Total ARGs**						
Category	3	0.27689	0.09230	86.863	5.49e-14***	
*Calves*						a
*Heifers*						b
*Lactating cows*						b
*Dry cows*						b
Farm	9	0.01835	0.00204	1.919	0.092	
*bla*_TEM_						
Category	3	0.00094430	0.00031477	4.5462	0.01052*	
*Calves*						a
*Heifers*						b
*Lactating cows*						b
*Dry cows*						ab
Farm	9	0.00064748	7.1942e-05	1.0391	0.43603	
*erm*B						
Category	3	0.088835	0.0296117	68.0679	1.032e-12***	
*Calves*						a
*Heifers*						b
*Lactating cows*						b
*Dry cows*						b
Farm	9	0.003335	0.0003705	0.8517	0.5772	
*sul*2						
Category	3	0.179038	0.059679	44.0924	1.536e-10***	
*Calves*						a
*Heifers*						b
*Lactating cows*						b
*Dry cows*						b
Farm	9	0.025206	0.002801	2.0692	0.06977	

**Figure 3:**
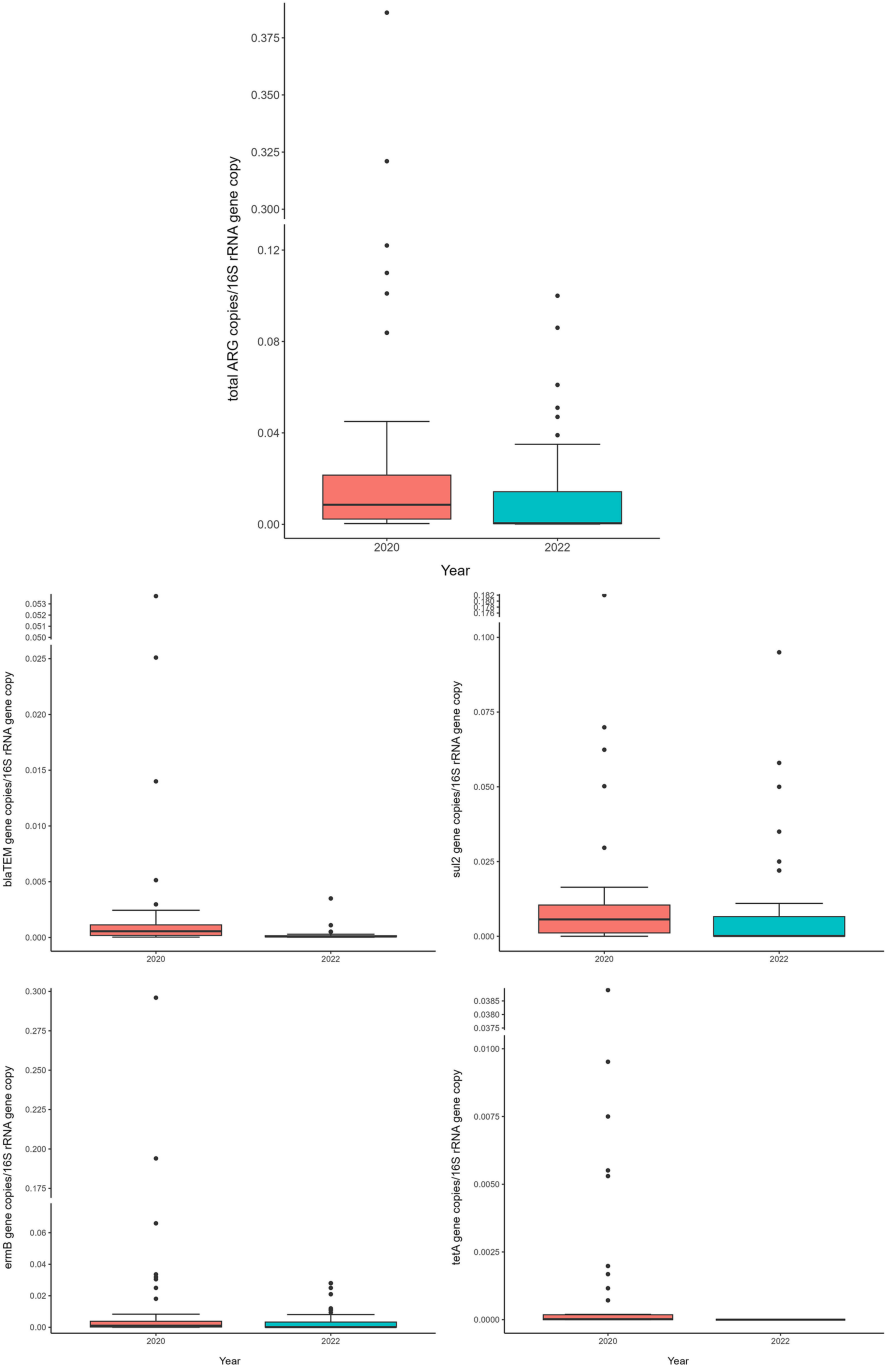
Normalized abundances of genes according to sampling year. Boxplots of the distribution of abundances of total ARGs, *bla*_TEM_, *erm*B, *sul*2 and *tet*A genes within the bacterial communities of 2020 and 2022. The thick horizontal line represents the median, the box represents 50% of the values, the whiskers extend to the highest and lowest value within the 1.5 interquartile range, dots represent the outliers.

**Figure 4:**
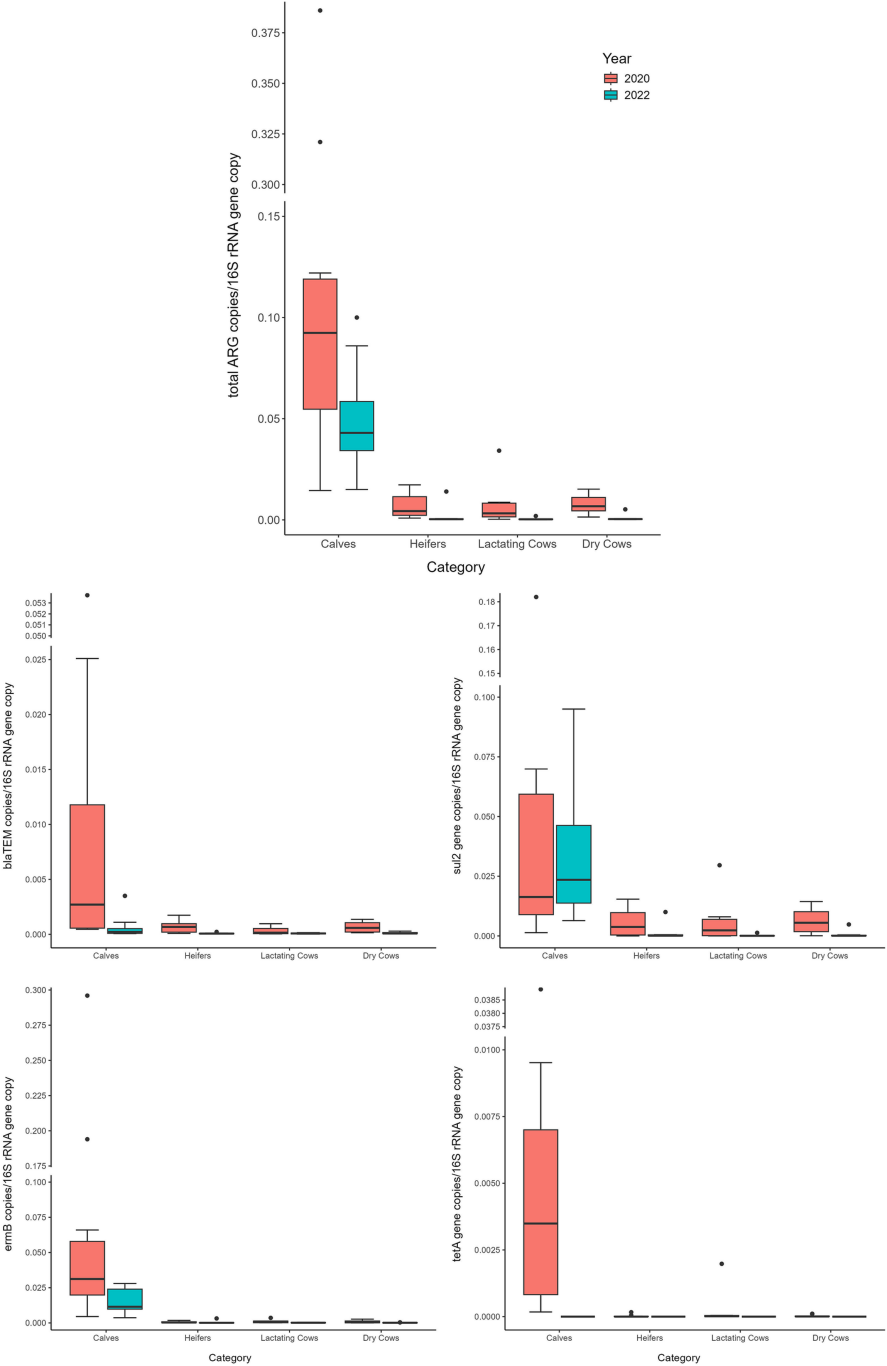
Normalized abundances of genes according to animal category and sampling year. Boxplots of the distribution of abundances of total ARGs, *bla*_TEM_, *erm*B, *sul*2 and *tet*A genes within the bacterial communities of 2020 and 2022 divided per farming category. The thick horizontal line represents the median, the box represents 50% of the values, the whiskers extend to the highest and lowest value within the 1.5 interquartile range, dots represent the outliers.

Comparing 2020 and 2022 data, the two sampling campaigns significantly differed (MANOVA: *p* = 0.0205). Indeed, there was a significant decrease, from 2020 to 2022, in the total ARG load (ANOVA: *p* ≤ 0.0118) (Figure 3 and Supplementary Table 4). In particular, the *bla*_TEM_ gene had a significantly lower abundance in 2022, in respect to 2020, and the *tet*A gene resulted no more detectable in 2022 (ANOVA: *p* ≤ 0.0019) (Figure 3 and Supplementary Table 4). No significant differences were found, according to the year, for the other investigated genes (ANOVA: *p* ≥ 0.0676), showing a comparable abundance in 2020 and 2022 (Figure 3 and Supplementary Table 4). When dividing samples per category, we observed a significant and generalized decrease of ARGs (single and total abundances) for DC in 2022 (ANOVA: *p* ≤ 0.0203) (Figure 4 and Supplementary Table 5). For the other categories, we found a significant reduction in total ARG load of 2022 (ANOVA: *p* ≤ 0.0457), driven by different genes according to the farming step: for LC, *bla*_TEM_, *erm*B, and *sul*2 genes had a significant lower abundance in 2022 (ANOVA: *p* ≤ 0.0367); for H, *bla*_TEM_, and *sul*2 genes significantly decreased in 2022 (ANOVA: *p* ≤ 0.0463); for C, *bla*_TEM_ and *tet*A genes were less abundant in 2022 (ANOVA: *p* ≤ 0.0226) (Figure 4 and Supplementary Table 5).

The data about the annual antimicrobial consumption referred to the year 2021 are shown in Supplementary Table 6. The consumption of single and total antibiotics did not significantly change over the time (MANOVA: *p* = 0.413; ANOVA: *p* ≥ 0.0748) (Supplementary Figure 1 and Supplementary Table 7).

### Correlation between ARG abundances and antibiotic consumption

The Pearson’s correlation analysis revealed a lack of correlation between the total normalized abundance of ARGs and the total consumption of antibiotics (*r* = −0.12, *p* = 0.7348) (Supplementary Table 8). Likewise, single genes (*bla*_TEM_, *erm*B, and *sul*2) and the corresponding antibiotics (penicillins, MLS, and sulphonamides) were not correlated (Supplementary Table 8).

## Discussion

The genes *erm*B and *bla*_TEM_, both classified as rank I ARGs (highest risk) according to the risk ranking for human health (14) were constitutively quantified in the sampled farms suggesting that the bred animals could be carriers of potentially antibiotic resistant bacteria and posing a threat to human health. This result is not surprising, considering that high-risk ARGs are commonly detected in the feces of food-producing animals (4, 15).

However, it is interesting to note that in the previous sampling campaign, among the quantified genes in the same farms, *erm*B resulted to be the most abundant gene (4); whereas, in the current sampling campaign, it ranked at the second place, with the *sul*2 gene being detected as the most abundant. Although limited to a selection of a few ARGs, this result could be interpreted as a positive indicator regarding potential concerns for human health, given the widespread of *sul*2 in environments (16, 17) including the pristine ones (18). Noteworthy, the *bla*_TEM_ gene abundance was significantly lower in the current sampling campaign if compared with the previous one. As this ARG is one of the most clinically relevant *β*-lactamases (19) and has been extensively detected in pathogenic bacteria isolated from food-producing animals (20, 21), its temporal reduction suggests a clear positive indication. Besides to these clinically relevant ARGs, *tet*A, previously identified as particularly abundant in the resistome of microbial communities isolated from fecal samples of some food-producing animals and negligible in the human gut (22), and not classified among the high-risk ARGs for human health (14) was not detected in the collected samples. This finding reinforces the observed trend of reduced ARGs over the sampling period.

Extending the analysis to the total ARG abundance and comparing the two sampling campaigns, it resulted significantly lower in 2022. This finding aligns perfectly with the results of the single ARGs as discussed above and underscores the success of the measures adopted to contrast the selection and spread of antibiotic resistance.

Interestingly, both single and total antibiotics prescribed in 2021 (2 years after the adoption of the guideline measures) were found to be similar to those used in 2019 (prior to the adoption of these measures). This could be due to the difference between antimicrobials prescribed and antimicrobials actually administered. Indeed, the farms included in the research were authorized by the Veterinary Authority to stock prescribed drugs (including antimicrobials) that can be administered when needed or disposed of when expired. For this reason, we cannot exclude that the amount of antimicrobials actually administered decreased since the first sampling. Furthermore, the quantity of antibiotics prescribed did not correlate with the ARG abundance in contrast to what previously found for the abundance of *bla*_TEM_ and the amount of penicillins used (4). This strongly suggests that the misuse of antibiotics [referring to inappropriate practices such as mass administration instead of the tailored approach recommended in the guideline (Table 1)] could significantly contribute to the selection and spread of antibiotic-resistant bacteria in food-producing animals, potentially posing a risk of transmission to humans (23).

Focusing on the dynamics of ARGs along the production chain, both, the total and single ARG abundances were significantly influenced by the sampling category rather than the individual sampled farm. This confirmed our previous results, where the “calves” category showed higher abundance of ARGs. Additionally, in this category, the temporal reduction observed over the two-year study period, while significant, was lower than that observed for the other farming stages. This finding strongly suggests that the early stage of cow life remains the phase that deserves more attention and possibly a dedicated strategy to contrast the spread of antibiotic resistance.

## Conclusion

Overall the obtained results encourage the adoption of (i) a rational use of antibiotics based on the diagnostic evidences and AST, (ii) enhance the biosecurity level, (iii) improve the hygiene practices with products targeted on the pathogens (parasites and bacteria) actually circulating in the farm, (iv) revising of the vaccine protocols for neonatal calf enteritis coupled with optimal on-farm colostrum management (v) vaccine strategies (auto-vaccine if commercial ones are not available) against mastitis sustained by contagious pathogens can significantly help in reducing the spread of antimicrobial resistance. Additionally, this study clearly identifies the most critical stage of the farming for the selection of ARGs, which confirms previous findings and directs future efforts toward tackling antibiotic resistance in the “calves” category. It could be beneficial to improve the quality of colostrum as it has previously been suggested as the main source of ARGs in the calf guts (24).

## Data Availability

The raw data supporting the conclusions of this article will be made available by the authors, without undue reservation.
