# Supplementary material for: The “best practices for farming” successfully contributed to decrease the antibiotic resistance gene abundances within dairy farms
Source: Front Vet Sci. 2025 Jan 7;11:1420282. doi: 10.3389/fvets.2024.1420282 (PMC11748548; doi:10.3389/fvets.2024.1420282)
Supplement: Supplementary file 1 [file Data_Sheet_1.docx]

**SUPPLEMENTARY FIGURES AND TABLES**


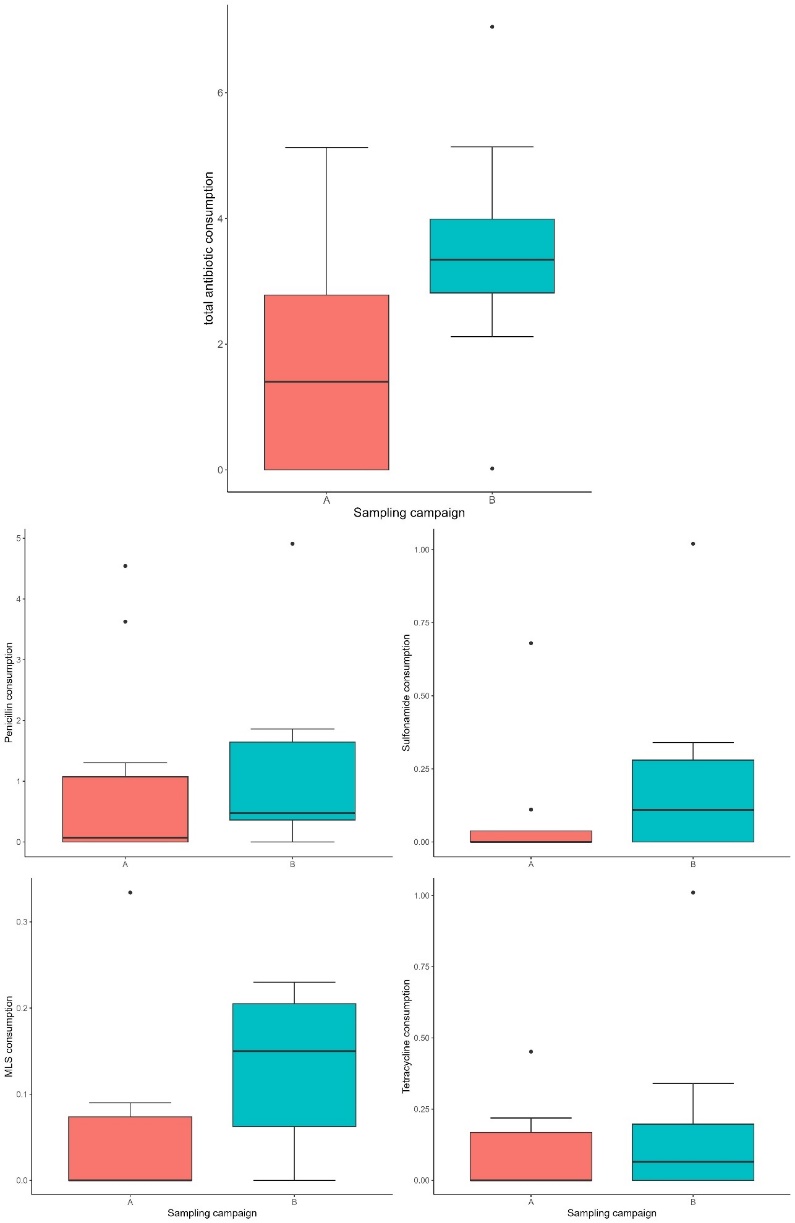


**Supplementary Figure 1. Antibiotic consumption according to sampling year.** Boxplots of the consumption of total antibiotics, penicillins, macrolides-lincosamides-streptogramins (MLS), sulfonamides and tetracyclines in the first (A) and second (B) sampling campaign. The thick horizontal line represents the median, the box represents 50% of the values, the whiskers extend to the highest and lowest value within the 1.5 interquartile range, dots represent the outliers.

**Supplementary Table 1. Sampled Farms.** Farm population and sampling size per animal category.

| **Farm** | **Herd population (Nr)** | **Category** | **Sampling size (Nr)** |
| --- | --- | --- | --- |
| A | >150 | Calves | 5 |
|  |  | Heifers | 10 |
|  |  | Lactating Cow | 10 |
|  |  | Dry Cow | 20 |
| B | >150 | Calves | 5 |
|  |  | Heifers | 10 |
|  |  | Lactating Cow | 10 |
|  |  | Dry Cow | 20 |
| C | <150 | Calves | 5 |
|  |  | Heifers | 5 |
|  |  | Lactating Cow | 5 |
|  |  | Dry Cow | 15 |
| D | >150 | Calves | 5 |
|  |  | Heifers | 10 |
|  |  | Lactating Cow | 10 |
|  |  | Dry Cow | 20 |
| E | >150 | Calves | 5 |
|  |  | Heifers | 10 |
|  |  | Lactating Cow | 10 |
|  |  | Dry Cow | 20 |
| F | >150 | Calves | 5 |
|  |  | Heifers | 10 |
|  |  | Lactating Cow | 10 |
|  |  | Dry Cow | 20 |
| G | <150 | Calves | 5 |
|  |  | Heifers | 5 |
|  |  | Lactating Cow | 5 |
|  |  | Dry Cow | 15 |
| H | <150 | Calves | 5 |
|  |  | Heifers | 5 |
|  |  | Lactating Cow | 5 |
|  |  | Dry Cow | 15 |
| I | >150 | Calves | 5 |
|  |  | Heifers | 10 |
|  |  | Lactating Cow | 10 |
|  |  | Dry Cow | 20 |
| J | >150 | Calves | 5 |
|  |  | Heifers | 10 |
|  |  | Lactating Cow | 10 |
|  |  | Dry Cow | 20 |

**Supplementary Table 2.**

ARG presence in the investigated farms (A-J) per productive category.

| Farms and categories | *bla*_CTXM_ | *bla*_TEM_ | *erm*B | *mcr*1 | *qnr*S | *sul*2 | *tet*A | *van*A |
| --- | --- | --- | --- | --- | --- | --- | --- | --- |
| A lacting cow | N | N | N | N | N | N | N | N |
| A dry cow | N | N | N | N | N | N | N | N |
| A heifers | N | N | N | N | N | N | N | N |
| A calves | N | P | P | N | N | P | N | N |
| B lacting cow | N | N | N | N | N | N | N | N |
| B dry cow | N | N | N | N | N | N | N | N |
| B heifers | N | N | N | N | N | N | N | N |
| B calves | N | N | P | N | N | P | N | N |
| C lacting cow | N | N | N | N | N | N | N | N |
| C dry cow | N | N | N | N | N | N | N | N |
| C heifers | N | N | N | N | N | N | N | N |
| C calves | N | N | P | N | N | P | N | N |
| D lacting cow | N | N | N | N | N | N | N | N |
| D dry cow | N | N | N | N | N | N | N | N |
| D heifers | N | N | N | N | N | N | N | N |
| D calves | N | N | P | N | N | P | N | N |
| E lacting cow | N | N | N | N | N | N | N | N |
| E dry cow | N | N | N | N | N | N | N | N |
| E heifers | N | N | N | N | N | N | N | N |
| E calves | N | N | P | N | N | P | N | N |
| F lacting cow | N | N | N | N | N | N | N | N |
| F dry cow | N | N | N | N | N | N | N | N |
| F heifers | N | N | N | N | N | N | N | N |
| F calves | N | N | P | N | N | P | N | N |
| G lacting cow | N | N | N | N | N | N | N | N |
| G dry cow | N | N | N | N | N | N | N | N |
| G heifers | N | N | N | N | N | N | N | N |
| G calves | N | N | P | N | N | P | N | N |
| H lacting cow | N | N | N | N | N | N | N | N |
| H dry cow | N | N | N | N | N | N | N | N |
| H heifers | N | N | N | N | N | N | N | N |
| H calves | N | N | P | N | N | P | N | N |
| I lacting cow | N | N | N | N | N | N | N | N |
| I dry cow | N | N | N | N | N | N | N | N |
| I heifers | N | N | N | N | N | N | N | N |
| I calves | N | N | P | N | N | P | N | N |
| J lacting cow | N | N | N | N | N | N | N | N |
| J dry cow | N | N | N | N | N | N | N | N |
| J heifers | N | N | N | N | N | N | N | N |
| J calves | N | N | P | N | N | P | N | N |

**Supplementary Table 3.** Normalized abundances of ARGs. The abundance of ARGs in the collected samples was quantified by ddPCR and normalized as gene copies/16S rRNA gene copy. C= calves, H= heifers, LC= Lactating cows, DC= dry cows.

| Farm | Category | *bla*_CTXM_ | *erm*B | *sul*2 |
| --- | --- | --- | --- | --- |
| A | C | 1.1E-03 | 2.8E-02 | 5.8E-02 |
| A | H | 7.6E-05 | 1.2E-04 | 4.8E-05 |
| A | LC | 1.8E-05 | 1.4E-04 | 3.4E-05 |
| A | DC | 4.1E-05 | 1.2E-04 | 2.2E-05 |
| B | C | 6.0E-05 | 8.1E-03 | 7.1E-03 |
| B | H | 4.1E-05 | 1.4E-04 | 5.3E-05 |
| B | LC | 4.2E-05 | 1.2E-04 | 5.8E-05 |
| B | DC | 7.0E-05 | 2.0E-05 | 8.0E-05 |
| C | C | 5.2E-04 | 3.7E-03 | 3.5E-02 |
| C | H | 9.1E-05 | 4.0E-05 | 5.2E-04 |
| C | LC | 1.5E-04 | 3.0E-04 | 3.0E-05 |
| C | DC | 8.6E-05 | 1.5E-04 | 5.3E-05 |
| D | C | 3.5E-03 | 2.5E-02 | 6.4E-03 |
| D | H | 6.9E-05 | 1.3E-04 | 3.9E-05 |
| D | LC | 6.4E-05 | 1.7E-04 | 7.5E-05 |
| D | DC | 1.9E-04 | 4.5E-05 | 0.0E+00 |
| E | C | 8.5E-05 | 2.1E-02 | 2.5E-02 |
| E | H | 4.7E-05 | 3.6E-05 | 2.4E-05 |
| E | LC | 5.0E-05 | 3.7E-04 | 2.2E-05 |
| E | DC | 5.5E-05 | 3.9E-04 | 3.2E-05 |
| F | C | 1.1E-04 | 1.1E-02 | 1.1E-02 |
| F | H | 2.9E-05 | 0.0E+00 | 4.6E-04 |
| F | LC | 1.2E-04 | 6.2E-05 | 8.1E-05 |
| F | DC | 2.9E-04 | 4.8E-05 | 4.8E-03 |
| G | C | 2.3E-04 | 1.2E-02 | 2.2E-02 |
| G | H | 1.1E-04 | 1.6E-04 | 3.6E-05 |
| G | LC | 1.1E-04 | 8.6E-05 | 8.5E-05 |
| G | DC | 5.6E-05 | 3.8E-04 | 1.3E-04 |
| H | C | 2.1E-04 | 2.8E-02 | 2.2E-02 |
| H | H | 2.2E-04 | 0.0E+00 | 1.5E-04 |
| H | LC | 1.4E-04 | 1.1E-04 | 6.0E-05 |
| H | DC | 1.3E-04 | 1.5E-04 | 7.2E-05 |
| I | C | 5.0E-04 | 1.1E-02 | 5.0E-02 |
| I | H | 5.7E-05 | 4.9E-05 | 4.3E-04 |
| I | LC | 2.9E-05 | 4.6E-05 | 2.2E-05 |
| I | DC | 6.5E-05 | 1.1E-04 | 2.9E-04 |
| J | C | 6.5E-05 | 9.4E-03 | 9.5E-02 |
| J | H | 6.1E-05 | 3.2E-03 | 1.0E-02 |
| J | LC | 9.5E-05 | 4.6E-04 | 1.3E-03 |
| J | DC | 1.7E-04 | 4.1E-05 | 4.4E-04 |

**Supplementary Table 4.** Statistical results for the analysis of variance (ANOVA) assessing the influence of the sampling year on the normalized abundance of total ARGs, *bla*_TEM_, *erm*B, *sul*2, and *tet*A genes.

|  | **Df** | **Sum Sq** | **Mean Sq** | **F-value** | **p-value** |
| --- | --- | --- | --- | --- | --- |
| **Total ARGs** |  |  |  |  |  |
| Year | 1 | 0.0987 | 0.0987 | 6.65 | 0.0118 * |
| ***bla*_TEM_** |  |  |  |  |  |
| Year | 1 | 0.010931 | 0.010931 | 11.079 | 0.001335 ** |
| ***erm*B** |  |  |  |  |  |
| Year | 1 | 0.02591 | 0.025912 | 3.0581 | 0.08427 |
| ***sul*2** |  |  |  |  |  |
| Year | 1 | 0.025206 | 0.002801 | 2.0692 | 0.06977 |
| ***tet*A** |  |  |  |  |  |
| Year | 1 | 0.007750 | 0.007750 | 10.357 | 0.00188 ** |

**Supplementary Table 5.** Statistical results for the analysis of variance (ANOVA) assessing the influence of the sampling year on the normalized abundance of total ARGs, *bla*_TEM_, *erm*B, *sul*2, and *tet*A genes according to the animal category.

|  |  | **Df** | **Sum Sq** | **Mean Sq** | **F-value** | **p-value** |
| --- | --- | --- | --- | --- | --- | --- |
| **Calves** | **Total ARGs** |  |  |  |  |  |
|  | Year | 1 | 0.07756 | 0.07756 | 4.606 | 0.0457 * |
|  | ***bla*_TEM_** |  |  |  |  |  |
|  | Year | 1 | 0.016656 | 0.016656 | 6.2149 | 0.02264 * |
|  | ***erm*B** |  |  |  |  |  |
|  | Year | 1 | 0.057753 | 0.057753 | 4.1454 | 0.05674 |
|  | ***sul*2** |  |  |  |  |  |
|  | Year | 1 | 0.000019 | 0.000019 | 0.0018 | 0.9665 |
|  | ***tet*A** |  |  |  |  |  |
|  | Year | 1 | 0.021718 | 0.021718 | 14.062 | 0.001466 ** |
| **Heifers** | **Total ARGs** |  |  |  |  |  |
|  | Year | 1 | 0.01098 | 0.01098 | 9.748 | 0.00589 ** |
|  | ***bla*_TEM_** |  |  |  |  |  |
|  | Year | 1 | 0.0011862 | 0.0011862 | 17.743 | 0.0005238 *** |
|  | ***erm*B** |  |  |  |  |  |
|  | Year | 1 | 0.0003067 | 0.0003067 | 1.542 | 0.2303 |
|  | ***sul*2** |  |  |  |  |  |
|  | Year | 1 | 0.0071278 | 0.0071278 | 4.5786 | 0.04632 * |
|  | ***tet*A** |  |  |  |  |  |
|  | Year | 1 | 2.9629e-05 | 2.9629e-05 | 3.0088 | 0.09991 |
| **Lactating cows** | **Total ARGs** |  |  |  |  |  |
|  | Year | 1 | 0.01316 | 0.01316 | 10.83 | 0.00406 ** |
|  | ***bla*_TEM_** |  |  |  |  |  |
|  | Year | 1 | 0.00023972 | 0.00023973 | 5.0918 | 0.03671 * |
|  | ***erm*B** |  |  |  |  |  |
|  | Year | 1 | 0.0011514 | 0.0011514 | 8.3141 | 0.009893 ** |
|  | ***sul*2** |  |  |  |  |  |
|  | Year | 1 | 0.009658 | 0.009658 | 6.4079 | 0.0209 * |
|  | ***tet*A** |  |  |  |  |  |
|  | Year | 1 | 0.00025227 | 0.00025227 | 2.7898 | 0.1122 |
| **Dry cows** | **Total ARGs** |  |  |  |  |  |
|  | Year | 1 | 0.01699 | 0.01699 | 29.31 | 3.82e-05 *** |
|  | ***bla*_TEM_** |  |  |  |  |  |
|  | Year | 1 | 0.00090722 | 0.00090722 | 16.574 | 0.0007169 *** |
|  | ***erm*B** |  |  |  |  |  |
|  | Year | 1 | 0.00091081 | 0.00091081 | 6.8279 | 0.01761 * |
|  | ***sul*2** |  |  |  |  |  |
|  | Year | 1 | 0.014406 | 0.014406 | 15.503 | 0.0009656 *** |
|  | ***tet*A** |  |  |  |  |  |
|  | Year | 1 | 5.4291e-05 | 5.4291e-05 | 6.4793 | 0.02029 * |

**Supplementary Table 6.** Annual antimicrobial consumption referred to 2021 of enrolled farms

| **Farm** | **Penicillins** | **MLS*** | **Sulfonamides** | **Tetracyclines** | **Total antibiotics** |
| --- | --- | --- | --- | --- | --- |
| A | 0.36 | 0.22 | 0.34 | 0.34 | 2.78 |
| B | 0.54 | 0.16 | 0 | 1.01 | 5.14 |
| C | 0.37 | 0.1 | 0 | 0.21 | 2.92 |
| D | 0.41 | 0.05 | 0.16 | 0 | 3.49 |
| E | 4.91 | 0.21 | 1.02 | 0 | 7.05 |
| F | 1.24 | 0 | 0.06 | 0.08 | 3.2 |
| G | 0.1 | 0.19 | 0.22 | 0.16 | 2.12 |
| H | 1.86 | 0.23 | 0.3 | 0.05 | 3.84 |
| I | 0 | 0 | 0 | 0 | 0.02 |
| J | 1.78 | 0.14 | 0 | 0 | 4.04 |

Data are expressed in Defined Daily Doses Animal for Italy (DDDAit) and extrapolated directly from the Classification of Intensive Animal Farming (ClassyFarm) information system based on the veterinary electronic prescription. Antimicrobials were categorized according to the World Health Organization (WHO). The total amount of antimicrobials includes all the active prescribed molecules used in the stable. *MLS, macrolides-lincosamides-streptogramins.

**Supplementary Table 7.** Statistical results for the analysis of variance (ANOVA) assessing the influence of the sampling year on the consumption of total antibiotics, penicillins, macrolides-lincosamides-streptogramins (MLS), sulfonamides, and tetracyclines.

|  | **Df** | **Sum Sq** | **Mean Sq** | **F-value** | **p-value** |
| --- | --- | --- | --- | --- | --- |
| **Total antibiotics** |  |  |  |  |  |
| Year | 1 | 0.517 | 0.517 | 1.079 | 0.313 |
| **Penicillins** |  |  |  |  |  |
| Year | 1 | 0.8126 | 0.8126 | 3.578 | 0.07475 |
| **MLS** |  |  |  |  |  |
| Year | 1 | 0.006784 | 0.006785 | 1.3206 | 0.2655 |
| **Sulfonamides** |  |  |  |  |  |
| Year | 1 | 0.00010 | 0.00010 | 0.0058 | 0.9399 |
| **Tetracyclines** |  |  |  |  |  |
| Year | 1 | 0.01365 | 0.01365 | 0.4203 | 0.525 |

**Supplementary Table 8.** Correlation between the abundance of ARGs and the consumption of antibiotics. MLS= macrolides-lincosamides-streptogramins

| **Pairwise** |  | **r** | **p-value** |
| --- | --- | --- | --- |
| Total ARGs | - total antibiotics | -0.1231 | 0.7348 |
| *bla*_TEM_ | - penicillins | -0.3010 | 0.398 |
| *erm*B | - MLS | 0.4633 | 0.1774 |
| *sul*2 | - sulphonamides | -0.1725 | 0.6336 |
